# Ileal pouch-anal anastomosis in familial adenomatous polyposis and ulcerative colitis with coexisting colorectal cancer: a guideline for treatment

**DOI:** 10.1093/jscr/rjaf332

**Published:** 2025-05-22

**Authors:** Mohammed Aldakhil, Nasser Alsanea

**Affiliations:** Surgery Department, College of Medicine, Qassim University, Al’Qassim-Saudi Arabia; Surgery Department, College of Medicine, Princess Nourah Bint Abdulrahman University, Riyadh, Saudi Arabia

**Keywords:** familial polyposis coli, ulcerative colitis, colorectal cancer, ileal pouch-anal anastomosis, total proctocolectomy, coexisting colorectal cancer

## Abstract

Patients with familial adenomatous polyposis (FAP) or ulcerative colitis (UC) are at increased risk of colorectal cancer (CRC), making surgical decision-making complex. This case series reviews six patients with FAP or UC who developed CRC and underwent ileal pouch-anal anastomosis (IPAA). Two patients with stage III CRC developed metastases, while the remaining four with stage I/II had no recurrence. IPAA is feasible in stage I/II CRC regardless of tumor location. For stage III colon cancer, total proctocolectomy (TPC) with IPAA followed by adjuvant chemotherapy is appropriate. In stage III rectal cancer more than 2 cm from the dentate line, IPAA can follow total neoadjuvant treatment (TNT). For tumors within 2 cm of the dentate line, TPC with end ileostomy is advised. IPAA may be contraindicated in cases with severe proctitis due to bleeding risk or radiation intolerance. Overall, IPAA is a suitable option for selected FAP or UC patients with CRC.

## Introduction

Among the relative contraindications for performing an ileal pouch-anal anastomosis (IPAA) is the presence of colorectal cancer (CRC) in patients with ulcerative colitis (UC) or familial adenomatous polyposis (FAP). This contraindication is particularly significant in cases of rectal cancer. In such scenarios, patients may require radiation therapy, and creating an ileal pouch would render it nonfunctional. Additionally, administering chemotherapy in the presence of a diverting ileostomy can be complicated by mucositis and a high-output stoma, which may lead to acute pre-renal failure.

Another concern is the risk of local pelvic recurrence in patients with an existing pouch, as this poses significant challenges for pouch excision. Previous studies report that 1–17% of these patients are diagnosed with coexisting CRC at the time of IPAA construction [1–5]. However, data on recurrence rates following IPAA in the presence of CRC remain scarce [1–5].

In this case series, we discuss the outcomes of performing IPAA in the presence of CRC and outline the approach we have adopted to manage such cases in the future.

## Presentation of case

Out of 201 IPAA procedures performed at King Faisal Specialist Hospital and Research Center in Riyadh, Saudi Arabia, six patients were found to have CRC at the time of presentation with a diagnosis of either ulcerative colitis UC or FAP. The diagnosis of CRC was confirmed preoperatively through pathological examination. Among these cases, five patients (three males and two females) were diagnosed with FAP, while one male patient was diagnosed with UC.

The median age at surgery was 38 years (range: 16–45) for FAP patients, while the UC patient was 53 years old. The median interval between diagnosis and surgery was nine months for FAP patients and 36 months for the UC patient. The cancer was located in the rectum in three cases and in the colon in the other three cases. The stage at diagnosis is detailed in Table 1: two patients had stage III disease (T3N2M0), while the remaining four were evenly distributed between stage I (T2N0M0) and stage II (T3N0M0).

**Table 1:** Characteristics of the cases

**Diagnosis**	**Site of Cancer**	**TNM**	**Stage**	**Duration of Symptoms**	**Follow-up**	**Recurrence**
*MUC*	Rectum	T2N0M0	I	36 months	48 months	No
*FAP*	Rectum	T2N0M0	I	9 months	72 months	No
*FAP*	Colon	T3N0M0	II	16 months	30 months	No
*FAP*	Colon	T3N0M0	II	14 months	31 months	No
*FAP*	Rectum	T3N2M0	III	2 months	84 months	Distant metastasis
*FAP*	Colon	T3N2M0	III	10 days	46 months	Distant metastasis

Patients with stage I rectal cancer underwent total mesorectal excision of the rectum without requiring radiation or chemotherapy. Similarly, patients with stage II colon cancer received only surgical excision, with no chemotherapy administered. One patient with stage III rectal cancer underwent total proctocolectomy with an end ileostomy, along with suspension of the small bowel outside the pelvis using a sling made of absorbable mesh. This patient received neoadjuvant chemoradiation and adjuvant chemotherapy postoperatively. An IPAA pouch was created 18 months after completing treatment, but the patient developed distant metastasis, necessitating second-line chemotherapy. The treatment caused mucositis and a high frequency of bowel movements, which were somewhat alleviated with codeine.

The second stage III patient underwent a left colectomy with IPAA creation and a diverting ileostomy, followed by adjuvant chemotherapy. The ileostomy was closed upon completing chemotherapy. Both stage III patients eventually developed distant metastases and succumbed to the disease. The remaining four patients showed no recurrence during a median follow-up period of 46 months. As indicated in Table 1, recurrence was confined to stage III patients, with these two cases also showing the shortest intervals from diagnosis to surgery.

## Discussion

Among patients with FAP, it is well-documented that cancer may be identified in the resected specimen [1–3]. This is likely due to the difficulty in diagnosing hidden cancer amidst the carpet of polyps observed during colonoscopy. A similar scenario applies to patients with UC [6]. Preoperative diagnosis of CRC is crucial, as it allows the surgeon to devise an optimal treatment plan. Conversely, discovering CRC intraoperatively may result in an improvised or suboptimal approach.

### Colon cancer

In cases of FAP or ulcerative colitis UC stage I/II, total proctocolectomy (TPC) with total mesocolic excision and PAA can be performed, as adjuvant chemotherapy is not required and the likelihood of recurrence is low [7]. For stage III FAP cases, adjuvant chemotherapy can be administered postoperatively. However, in stage III UC, there is an 82% chance of grade III/IV diarrhea [8]. In such scenarios, the optimal treatment involves the infusion-based administration of 5-FU and oxaliplatin (FOLFOX), while avoiding bolus administration and irinotecan, as both exacerbate diarrhea [9]. Additionally, aminosalicylates can help alleviate diarrhea symptoms [8].

### Rectal cancer

In cases of stage I/II rectal cancer associated with UC or FAP, concurrent TPC and IPAA can be performed, as there is no need for adjuvant treatment and the chances of recurrence are low.

For stage III FAP or UC with rectal cancer located more than 2 cm cephalad to the dentate line, patients should undergo total neoadjuvant treatment (TNT), consisting of induction chemotherapy with FOLFOX followed by consolidation chemoradiation [10], prior to concurrent TPC and IPAA. Conversely, in cases where rectal cancer is located within 2 cm of the dentate line, TPC with removal of the anus and the creation of an end ileostomy is recommended [11].

An alternative approach for stage III rectal cancer in UC involves removing the rectum (if the tumor is in the upper or middle rectum) along with total colectomy, while keeping the small bowel outside the pelvis using a sling made of biological mesh or an inflatable silicone prosthesis [12]. The biological mesh is preferred, as it avoids the placement of foreign material in the pelvis, which is especially beneficial for immunocompromised patients. After completing chemoradiation, a concurrent completion proctectomy is performed along with IPAA. However, delivering radiation postoperatively, after cancer resection, is less effective. Patients must also be informed of the rare risk of massive rectal bleeding, which could necessitate emergency surgery [9]. If a patient cannot tolerate radiation therapy, TPC with end ileostomy remains a viable option.

The severity of proctitis dictates the timing of radiation. If severe, the only option may be to administer adjuvant chemoradiation after total mesorectal excision of the rectum and colon, with the creation of an ileostomy [11]. It is critical to avoid irradiating an ileal pouch, as this is associated with a 16% risk of pouch failure [3].

## Conclusion

IPAA is a viable surgical option for patients with FAP and UC. Factors influencing the treatment plan and surgical approach include the underlying diagnosis (FAP or UC), the stage of CRC, the need for chemotherapy or radiation, and the severity of mucositis.

For stage I/II CRC in both FAP and UC, as well as stage III colon cancer in FAP and UC, upfront concurrent TPC and IPAA can be performed. In contrast, stage III rectal cancer in FAP and UC should be managed with TNT followed by IPAA.

It is important to note the rare risk of massive bleeding in cases of severe proctitis associated with UC subjected to radiation. Additionally, radiation therapy involving the ileal pouch should be avoided, as it carries a significant risk of failure.

## Limitations

This case series has several important limitations. First, the small sample size may restrict the generalizability of the findings to a wider population of patients with FAP or UC complicated by synchronous colorectal cancer. Second, the retrospective nature of the study introduces inherent biases, such as incomplete documentation or selective data collection [13]. Despite these limitations, this study provides a valuable contribution to the limited body of literature on managing patients with dual diagnoses of inherited or inflammatory colorectal conditions and synchronous colorectal cancer. It offers preliminary guidance for multidisciplinary treatment planning.
